# Comparison of blood-based liver fibrosis scores in the Mount Sinai Health System, MASLD Registry, and NHANES 2017–2020 study

**DOI:** 10.1097/HC9.0000000000000515

**Published:** 2024-08-26

**Authors:** Robert Chen, Ben Omega Petrazzini, Girish Nadkarni, Ghislain Rocheleau, Meena B. Bansal, Ron Do

**Affiliations:** 1 The Charles Bronfman Institute for Personalized Medicine, Icahn School of Medicine at Mount Sinai, New York, New York, USA; 2 Department of Genetics and Genomic Sciences, Icahn School of Medicine at Mount Sinai, New York, New York, USA; 3 Medical Scientist Training Program, Icahn School of Medicine at Mount Sinai, New York, New York, USA; 4 Center for Genomic Data Analytics, Icahn School of Medicine at Mount Sinai, New York, New York, USA; 5 Division of Liver Diseases, Department of Medicine, Icahn School of Medicine at Mount Sinai, New York, New York, USA

## Abstract

**Background:**

Liver fibrosis is a critical public health concern, necessitating early detection to prevent progression. This study evaluates the recently developed LiverRisk score and steatosis-associated Fibrosis Estimator (SAFE) score against established indices for prognostication and/or fibrosis prediction in 4diverse cohorts, including participants with metabolic dysfunction–associated steatotic liver disease (MASLD).

**Methods:**

We used data from the Mount Sinai Data Warehouse (32,828 participants without liver disease diagnoses), the Mount Sinai MASLD/MASH Longitudinal Registry (422 participants with MASLD), and National Health and Nutrition Examination Survey 2017–2020 (4133 participants representing the general population) to compare LiverRisk score, FIB-4 index, APRI, and SAFE score. Analyses included Cox proportional hazards regressions, Kaplan-Meier estimates, and classification metrics to evaluate performance in prognostication and fibrosis prediction.

**Results:**

In Mount Sinai Data Warehouse, LiverRisk score was significantly associated with future liver-related outcomes but did not significantly outperform FIB-4 or APRI for predicting any of the outcomes. In the general population, LiverRisk score and SAFE score outperformed FIB-4 and APRI in identifying fibrosis, but LiverRisk score underperformed among participants who were non-White or had type 2 diabetes. Among participants with MASLD, SAFE score outperformed FIB-4 and APRI in 1 of 2 cohorts, but there were generally few significant performance differences between all 4 scores.

**Conclusions:**

LiverRisk score does not consistently outperform existing predictors in diverse populations, and further validation is needed before adoption in settings with significant differences from the original derivation cohorts. It remains necessary to replicate the ability of these scores to predict liver-specific mortality, as well as to develop diagnostic tools for liver fibrosis that are accessible and substantially better than current scores, especially among patients with MASLD and other chronic liver conditions.

## Graphical abstract

**Figure:**
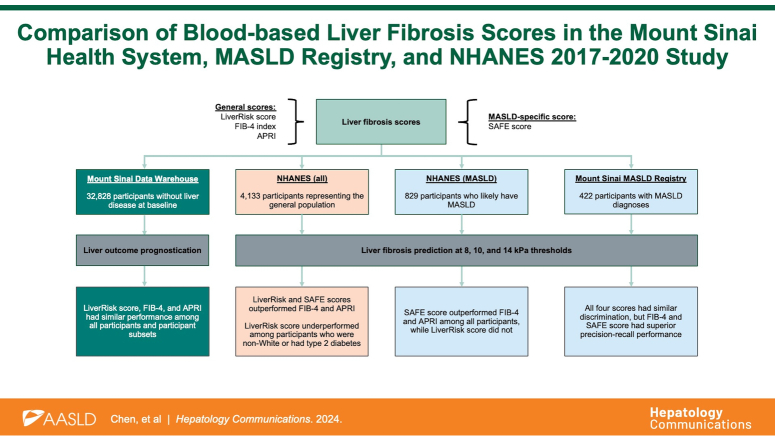

## INTRODUCTION

The global burden of liver disease, particularly liver fibrosis and its progression to cirrhosis and HCC, presents a substantial public health challenge. Early detection of liver fibrosis is critical for preventing disease progression yet remains challenging due to the asymptomatic nature of early-stage fibrosis and the limitations of current diagnostic tools. Liver biopsy is invasive, subject to sampling error, and has large interobserver variability. Elastography-based methods are reasonably accurate but not widely available, whereas existing blood-based tests like the fibrosis-4 index (FIB-4) and aspartate aminotransferase (AST) to Platelet Ratio Index (APRI) have limited accuracy to rule in significant fibrosis.^1,2^

The LiverRisk score, derived by Serra-Burriel et al^3^ from a prospective international cohort study, is a novel, noninvasive approach for identifying individuals at risk of liver-related morbidity and mortality based on age, sex, and 6 laboratory measurements. Initial validation of the LiverRisk score in 2 cohorts demonstrated superiority in predicting liver stiffness and future liver-related outcomes compared to FIB-4 index and APRI. Importantly, the derivation and validation cohorts were comprised primarily of White European individuals, and prognostic evaluation was performed only in the UK Biobank, which is subject to a healthy volunteer selection bias.^4^ Thus, it is unknown whether LiverRisk score is generalizable to other ethnic groups, health care settings, and individuals with metabolic dysfunction–associated steatotic liver disease (MASLD), currently the most common cause of chronic liver disease.^5^ It is also unknown how LiverRisk score compares to steatosis-associated Fibrosis Estimator (SAFE) score,^6^ a novel MASLD-specific fibrosis score.

In this study, we address these limitations by conducting a comparison of LiverRisk score, FIB-4 index, APRI, and SAFE score among large, racially and ethnically diverse cohorts, including 32,828 participants without liver disease diagnoses from the Mount Sinai Data Warehouse (MSDW), 422 participants with clinical MASLD diagnoses from the Mount Sinai MASLD/ metabolic dysfunction–associated steatohepatitis Longitudinal Registry (MASLD Registry), and 4133 participants from the National Health and Nutrition Examination Survey (NHANES) 2017–2020 cycle, of whom 829 likely have MASLD. MSDW permits prognostic evaluation in a real-world health care population with substantially different demographic composition compared to the initial LiverRisk score validation cohorts; NHANES permits a comparison of the scores among participants representative of the diverse US population; and the MASLD Registry permits a focused assessment of liver fibrosis prediction among patients with MASLD, which is important for monitoring disease progression. Using results from all cohorts, we provide insight into the use of blood-based tests to identify and manage individuals at risk for liver fibrosis.

## METHODS

### Study populations

We compared LiverRisk score, FIB-4 index, APRI, and SAFE score among participants from the Mount Sinai Health System and the NHANES 2017–2020 cycle. We evaluated prognostication among a retrospective cohort of 32,828 nonhospitalized participants from Mount Sinai Data Warehouse (MSDW) without liver disease at baseline. We evaluated fibrosis prediction among 3 cross-sectional cohorts: a cohort of all eligible NHANES participants (NHANES [all]; n=4133), a subcohort of NHANES participants who likely have MASLD (NHANES [MASLD]; n=829), and a cohort of 422 participants with MASLD diagnoses from the Mount Sinai MASLD Registry. All research was conducted in accordance with both the Declarations of Helsinki and Istanbul; research in MSDW was exempt from IRB review as all data was deidentified, while use of the MASLD Registry in this study was approved by the Icahn School of Medicine Institutional Review Board under study 22-00080. All participants in the MASLD Registry provided written consent.

MSDW consists of Epic-derived electronic health records for more than 11 million patients from 6 facilities across the Mount Sinai Health System (Mount Sinai Hospital, Mount Sinai Queens, Mount Sinai West, Mount Sinai Morningside, Mount Sinai Brooklyn, Mount Sinai Beth Israel). From MSDW, we identified 49,224 patients with alanine aminotransferase (ALT), AST, gamma-glutamyl transferase (GGT), and platelet measurements available from any single health care encounter between January 1, 2000, and December 31, 2019, as well as random glucose and cholesterol measurements within 1 year prior to this baseline date. Although fasting glucose measurements were unavailable, our use of random glucose is consistent with Serra-Burriel and colleagues’ initial validation in the UK Biobank, which also used random glucose measurements. For patients with complete measurements available from more than one encounter, we used the earliest encounter as the baseline to maximize follow-up time. Of the 49,224 patients, 46,567 were between 19 and 92 years of age, 42,547 did not have exclusionary diagnoses, and 32,828 were not hospitalized at baseline. We did not assess SAFE score in MSDW due to the limited availability of globulin (ie, albumin—total protein) measurements and because of its intended specificity for patients with MASLD.

The MASLD Registry represents 699 pediatric and adult participants with a clinical MASLD diagnosis being followed at the outpatient liver clinic of any Mount Sinai Health System hospital; 546 of these participants have received transient elastography (FibroScan) measurements as of March 1, 2024. All participants have alcohol consumption below 140 g/wk for women and 210 g/wk for men. In this study, we included 422 of the 546 participants who were between 19 and 92 years of age at the date of their FibroScan measurement and who had all 6 LiverRisk score measurements available within an 18-month window containing this date.

The NHANES 2017–2020 study includes 15,560 participants from across the United States, of whom 9023 had complete transient elastography measurements. A total of 7577 of these participants were between 19 and 92 years of age. We included 4557 of these participants who had all 6 LiverRisk score measurements available and who reported a fasting time ≥8 hours. We identified 829 of the 4557 participants as likely having MASLD using a controlled attenuation parameter score cutoff of 302 to define S1 steatosis, which in the study by Eddowes et al^7^ optimized sensitivity (0.80) and specificity (0.83). In this subcohort, we included only participants who reported consuming alcohol once a week or less frequently or who reported drinking one or fewer beverages on days they consumed alcohol. All 829 participants met cardiometabolic criteria for MASLD.

### Liver outcome and comorbidity definitions

We adopted the same definitions as Serra-Burriel et al^3^ for exclusionary diagnoses, liver outcomes, and liver-related hospitalization. Exclusionary diagnoses included international classification of diseases-10 codes B15-B19 at any time point or C22 and K70-K77 at baseline. Liver outcomes included “any liver outcome,” defined as codes C22.0, C22.7, C22.8, K70, K71.7, K71.9, K72.1, K72.9, K73, K74 (except K74.3 and K74.4), K75 (except K75.0, K75.3, and K75.4), and K76-K77; “liver-related hospitalization,” defined as the first inpatient encounter after the baseline date where any of the “any liver outcome” codes was assigned; and cirrhosis, defined as codes C22.0, K70.3, K70.4, K72.9, K74.5, K74.6, and K76.6 based on recommendations of Hayward et al.^8^ We did not perform mortality analyses due to a lack of complete mortality data in MSDW. We also did not examine liver cancer (codes C22.0, C22.7, and C22.8) as there were only 16 prospective events; we excluded most patients with liver cancer as they were diagnosed prior to or during their first encounter with all 6 LiverRisk score measurements available. We assessed the presence of nonliver comorbidities using international classification of diseases-10 codes included in the Charlson Comorbidity Index^9^ and type 2 diabetes using the international classification of diseases-10 code E11.

### Statistical analyses

We performed Cox proportional hazards regressions and Kaplan-Meier estimates using the Python lifelines (version 0.28.0) package. For Cox regressions, we adjusted for age, gender, and self-reported race/ethnicity, used the date of the most recent health care encounter as the censoring date, and estimated robust errors using the Huber sandwich estimator to account for time-covariate interactions.^10^

We calculated metrics using the Python scipy (version 1.12.0) and scikit-learn (version 1.4.1) packages. Except for the cutoff-dependent analyses, where we used the empirical bootstrap to reduce computation time, we calculated 95% CIs for all metrics using the bias-corrected and accelerated procedure with 1000 bootstraps. We compared predictor performance using paired permutation tests with 1000 iterations.^11^ We considered *p*<0.05 significant.

## RESULTS

### Study sample

All 4 cohorts included in this analysis (MSDW, NHANES [all], NHANES [MASLD], and MASLD Registry) were ethnically diverse, with 34.8%, 37.2%, 50.8%, and 18.1% of individuals in the cohorts identifying as non-Hispanic White, respectively (Table 1). The prevalence of F2-F4 fibrosis, defined as liver stiffness ≥8 kPa, was 9.6% in the NHANES (all) cohort, 21.6% in the NHANES (MASLD) cohort, and 37.7% in the MASLD Registry cohort.

**TABLE 1:** Composition of the 4 cohorts used in this study

Variable	MSDW	NHANES (all)	NHANES (MASLD)	MASLD registry
Total participants	32,828	4133	829	422
Demographics				
Age	52.6 [38.5–65.5]	51.0 [34.0–64.0]	54.0 [41.0–65.0]	58.7 [47.7–66.1]
Female	17,224 (52.5)	2343 (51.4)	359 (43.3)	237 (56.2)
Non-Hispanic White	11,422 (34.8)	1539 (37.2)	312 (50.8)	111 (18.1)
Non-Hispanic Black	5060 (15.4)	1183 (28.6)	243 (39.6)	37 (6.0)
Non-Hispanic Asian	998 (3.0)	541 (13.1)	154 (25.1)	30 (4.9)
Hispanic	5206 (15.9)	1046 (25.3)	78 (12.7)	223 (36.3)
Other/unspecified race	10,142 (30.9)	248 (6.0)	42 (6.8)	21 (3.4)
Nonliver comorbidity	15,283 (46.6)	Not assessed	Not assessed	422 (100.0)
Type 2 diabetes	5,393 (16.4)	693 (15.2)	243 (29.3)	221 (52.4)
Measurements				
Body mass index	27.2 [23.6–31.6]	28.7 [24.8–33.7]	33.9 [29.6–38.8]	31.3 [28.0–35.5]
Glucose (mmol/L)	5.1 [4.4–6.0]	5.3 [4.9–5.9]	5.8 [5.2–7.0]	5.3 [4.7–6.5]
Cholesterol (mmol/L)	4.6 [3.9–5.4]	4.7 [4.0–5.4]	4.7 [4.1–5.4]	4.6 [3.8–5.3]
AST (U/L)	23.0 [18.0–30.0]	19.0 [16.0–24.0]	20.0 [16.0–26.0]	30.0 [22.0–40.8]
ALT (U/L)	21.0 [15.0–32.0]	17.0 [13.0–25.0]	22.0 [16.0–33.0]	35.5 [24.0–54.0]
GGT (U/L)	24.0 [16.0–41.0]	21.0 [15.0–32.0]	26.0 [19.0–40.0]	46.0 [28.0–76.8]
Platelets (10^9^/L)	237.0 [196.0–284.0]	236.0 [199.0–279.0]	237.0 [202.0–279.0]	234.5 [183.8–287.8]
Globulin (g/dL)	Not assessed	3.1 [2.8–3.4]	3.1 [2.9–3.4]	3.0 [2.6–3.4]
Liver stiffness (kPa)	Not assessed	5.0 [4.1–6.1]	5.8 [4.7–7.5]	6.7 [4.8–10.9]
<8 (F1)	—	3,721 (90.4)	650 (78.4)	263 (62.3)
8–10 (F2)	—	186 (4.2)	71 (8.6)	35 (8.3)
>10–14 (F3)	—	122 (3.0)	64 (7.7)	52 (12.3)
≥14 (F4)	—	104 (2.5)	44 (5.3)	72 (17.1)
CAP score (dB/m)	Not assessed	262.0 [218.0–308.0]	337.0 [319.0–361.0]	305.0 [271.0–350.8]
<238 (S0)	—	1,499 (36.3)	0 (0.0)	43 (10.4)
238–260 (S1)	—	516 (12.5)	0 (0.0)	37 (8.9)
>260–290 (S2)	—	710 (17.2)	0 (0.0)	86 (20.8)
≥290 (S3)	—	1,407 (34.1)	829 (100.0)	248 (60.0)
Predictors				
LiverRisk score	5.3 [4.5–6.5]	5.1 [4.5–5.9]	5.7 [4.9–6.6]	6.1 [5.2–7.7]
FIB-4 index	1.1 [0.7–1.6]	0.9 [0.6–1.4]	0.9 [0.6–1.3]	1.2 [0.8–1.9]
APRI	0.2 [0.2–0.4]	0.2 [0.2–0.3]	0.2 [0.2–0.3]	0.3 [0.2–0.5]
SAFE score	Not assessed	−8.4 [−78.2 to 63.7]	30.1 [−30.9 to 93.0]	79.9 [−7.6 to 160.0]

*Note:* Values are either counts with (percentages) or medians with [interquartile ranges]. Nonliver comorbidities were defined as those included in the Charlson Comorbidity Index. NHANES (all) represents all eligible NHANES participants while NHANES (MASLD) represents the subset of NHANES participants who likely have MASLD.

Abbreviations: ALT, alanine aminotransferase; APRI, AST to Platelet Ratio Index; AST, aspartate aminotransferase; CAP, controlled attenuation parameter; FIB-4, fibrosis-4 index; GGT, gamma glutamyltransferase; MASLD, metabolic dysfunction–associated steatotic liver disease; MSDW, Mount Sinai Data Warehouse; NHANES, National Health and Nutrition Examination Survey; SAFE, steatosis-associated Fibrosis Estimator.

### Prognostication in a real-world health care population

**FIGURE 1:**
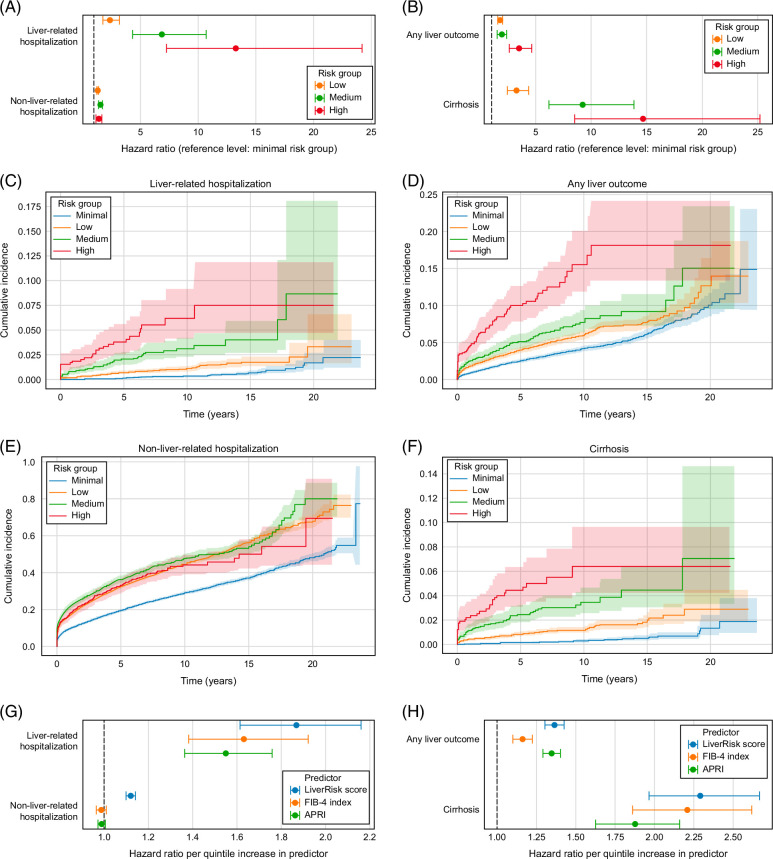
Prognostication among 32,828 Mount Sinai Data Warehouse participants without liver disease at baseline. (A, B) HRs with 95% CIs for liver-related hospitalization and nonliver-related hospitalization (A) or any liver outcome and cirrhosis (B) for participants in the low, medium, and high-risk groups of LiverRisk score compared to participants in the minimal-risk group. (C–F) Kaplan-Meier curves with 95% CIs showing the cumulative incidence of liver-related hospitalization (C), any liver outcome (D), nonliver-related hospitalization (E), and cirrhosis (F) for participants in the minimal, low, medium, and high-risk groups of LiverRisk score. (G–H) HRs with 95% CIs for liver-related hospitalization and nonliver-related hospitalization (G) or any liver outcome and cirrhosis (H) for each quintile increase in LiverRisk score, FIB-4 index, or APRI.

In the MSDW cohort, there were 22,102, 8,673, 1,512, and 541 participants in the minimal, low, medium, and high-risk groups for LiverRisk score, respectively. Median follow-up periods for liver-related hospitalization, nonliver-related hospitalization, any liver outcome, and cirrhosis were 6.3 (IQR 3.1–9.1), 5.3 (1.2–8.4), 6.1 (2.6–9.0), and 6.3 (3.1–9.1) years, respectively. Participants in the low-risk, medium-risk, and high-risk groups had significantly higher HRs for all 4 outcomes compared to those in the minimal-risk groups (Figure 1A–F, Supplemental Table S1, http://links.lww.com/HC9/B16). However, there was a continuous increase in HR from the low to high-risk groups only for the liver-related outcomes and not for nonliver-related hospitalization, replicating the specificity of LiverRisk score for liver-related morbidity. Among 541 participants in the high-risk group, there were HRs of 13.25 (95% CI: 7.26–24.2) for liver-related hospitalization, 3.46 (2.59–4.63) for any liver-related outcome, and 14.64 (8.52–25.18) for cirrhosis. The HR for liver-related hospitalization was significantly lower than that reported in the UK Biobank (>100).

For FIB-4 index, there were 20,562, 9475, 942, and 1849 participants in the <1.30, 1.30–2.67, >2.67–3.25, and >3.25 groups, respectively, while for APRI, there were 28,671, 2715, 538, and 904 participants in the <0.5, 0.5–1.0, >1.0–1.5, and >1.5 groups (Supplemental Table S1, http://links.lww.com/HC9/B16). Compared to LiverRisk score, both scores had similar event rates in the lowest-risk groups but had substantially more participants assigned to their respective highest-risk groups. Accordingly, for the highest-risk groups of FIB-4 index and APRI, HRs for liver-related hospitalization and any liver outcome were closer to those of the medium-risk rather than high-risk LiverRisk score group (Supplemental Figure S1, http://links.lww.com/HC9/B17; Supplemental Table S1, http://links.lww.com/HC9/B16). However, HRs for cirrhosis were approximately equal for participants in the high-risk LiverRisk score group, >3.25 FIB-4 index group, and >1.5 APRI group.

To directly compare LiverRisk score, FIB-4 index, and APRI, we partitioned participants into quintiles separately for each score and examined the HR per quintile increase for each outcome (Supplemental Table S2, http://links.lww.com/HC9/B16). Unlike the UK Biobank, we did not observe significant differences in HRs between the 3 scores for any of the outcomes, with all 3 scores being significantly associated with liver-related hospitalization, any liver outcome, and cirrhosis. Additionally, for nonliver-related hospitalization, neither FIB-4 index nor APRI had HRs significantly >1, suggesting specificity for liver-related outcomes is not specific to the LiverRisk score. These results were generally consistent across subsets of ethnicity, gender, and presence of nonliver comorbidities. However, HRs per quintile increase in LiverRisk score for any liver outcome were significantly larger for males compared to females (*p*=6.8×10^−4^) and non-Hispanic White compared to not non-Hispanic White participants (*p*=6.3×10^−6^) (Supplemental Table S2, http://links.lww.com/HC9/B16), whereas there were no significant differences for FIB-4 index for these comparisons (*p*=0.53 and 0.07, respectively). Likewise, for liver hospitalization and cirrhosis, there was an ns trend toward larger HRs per quintile increase in LiverRisk score among male and non-Hispanic White participants. Among participants who reported daily alcohol consumption, while LiverRisk score and APRI were significantly associated with all 3 liver-related outcomes, FIB-4 index was only significantly associated with cirrhosis.

Replicating the original LiverRisk score methodology, we also compared HRs for participants in the 90%–95%, 95%–99%, and ≥99% quantiles compared to the <90% quantile of LiverRisk score, FIB-4 index, or APRI (Supplemental Table S3, http://links.lww.com/HC9/B16). Here, LiverRisk score also did not have significantly different HRs compared to either FIB-4 index or APRI for any of the outcomes.

Multivariable Cox regressions of all LiverRisk score components with each outcome showed that besides age and GGT, none of the features was consistently associated with all outcomes (Supplemental Table S4, http://links.lww.com/HC9/B16). For example, AST was significantly associated with liver hospitalization (*p*=0.003) and cirrhosis (*p*=6.62×10^−6^) but not any liver outcome (*p*=0.06). Association of glucose, cholesterol, and GGT with FIB-4 index and of glucose, cholesterol, ALT, and GGT with APRI demonstrated that these features still had significant associations with at least one outcome, suggesting that LiverRisk score components missing in FIB-4 index and APRI are important for prognostication in MSDW.

### Fibrosis prediction in the general population

Among all eligible NHANES participants, LiverRisk score and SAFE score significantly outperformed FIB-4 index and APRI in both AUROC and area under the precision-recall curve (AUPRC) for distinguishing stiffness ≥8 and ≥10 kPa (hereafter referred to as classification tasks), while only SAFE score outperformed FIB-4 index and APRI in AUROC for the ≥14 kPa classification task (Table 2; Supplemental Tables S5, S6, http://links.lww.com/HC9/B16). However, there were no significant differences in AUROC between LiverRisk score and SAFE score for any of the 3 classification tasks, and SAFE score outperformed LiverRisk score in AUPRC for all 3 classification tasks.

**TABLE 2:** Performance metrics among all participants in each cohort

Predictor	Classification task (kPa)	Cases/group size (%)	AUROC (95% CI)	AUPRC (95% CI)
NHANES (all)				
LiverRisk score	≥8	412/4557 (9.0)	0.737 (0.71-0.763)	0.275 (0.228-0.313)
FIB-4 index	—	412/4557 (9.0)	0.621 (0.587-0.646)	0.2 (0.161-0.228)
APRI	—	412/4557 (9.0)	0.647 (0.617-0.672)	0.225 (0.186-0.26)
SAFE score	—	389/4355 (8.9)	0.748 (0.72-0.775)	0.293 (0.247-0.346)
LiverRisk score	≥10	226/4557 (5.0)	0.759 (0.721-0.79)	0.197 (0.15-0.237)
FIB-4 index	—	226/4557 (5.0)	0.677 (0.63-0.713)	0.181 (0.131-0.226)
APRI	—	226/4557 (5.0)	0.692 (0.652-0.727)	0.182 (0.131-0.223)
SAFE score	—	214/4355 (4.9)	0.802 (0.772-0.834)	0.268 (0.207-0.327)
LiverRisk score	≥14	104/4557 (2.3)	0.791 (0.743-0.833)	0.143 (0.085-0.187)
FIB-4 index	—	104/4557 (2.3)	0.727 (0.666-0.777)	0.177 (0.101-0.248)
APRI	—	104/4557 (2.3)	0.731 (0.673-0.781)	0.172 (0.101-0.238)
SAFE score	—	98/4355 (2.3)	0.841 (0.801-0.887)	0.261 (0.172-0.338)
NHANES (MASLD)				
LiverRisk score	≥8	179/829 (21.6)	0.675 (0.634-0.717)	0.398 (0.32-0.457)
FIB-4 index	—	179/829 (21.6)	0.58 (0.532-0.63)	0.321 (0.248-0.375)
APRI	—	179/829 (21.6)	0.616 (0.565-0.67)	0.375 (0.299-0.435)
SAFE score	—	168/782 (21.5)	0.693 (0.641-0.733)	0.435 (0.355-0.501)
LiverRisk score	≥10	108/829 (13.0)	0.668 (0.613-0.713)	0.237 (0.169-0.298)
FIB-4 index	—	108/829 (13.0)	0.614 (0.554-0.669)	0.224 (0.145-0.272)
APRI	—	108/829 (13.0)	0.623 (0.567-0.688)	0.239 (0.168-0.29)
SAFE score	—	103/782 (13.2)	0.73 (0.677-0.778)	0.332 (0.245-0.411)
LiverRisk score	≥14	44/829 (5.3)	0.659 (0.572-0.747)	0.12 (0.057-0.185)
FIB-4 index	—	44/829 (5.3)	0.605 (0.501-0.687)	0.113 (0.047-0.165)
APRI	—	44/829 (5.3)	0.652 (0.567-0.746)	0.128 (0.061-0.192)
SAFE score	—	42/782 (5.4)	0.733 (0.647-0.814)	0.208 (0.105-0.304)
MASLD Registry participants				
LiverRisk score	≥8	159/422 (37.7)	0.71 (0.652-0.759)	0.583 (0.511-0.667)
FIB-4 index	—	159/422 (37.7)	0.714 (0.664-0.765)	0.651 (0.579-0.721)
APRI	—	159/422 (37.7)	0.696 (0.639-0.747)	0.625 (0.542-0.683)
SAFE score	—	159/422 (37.7)	0.747 (0.695-0.794)	0.671 (0.581-0.734)
LiverRisk score	≥10	124/422 (29.4)	0.717 (0.66-0.774)	0.492 (0.401-0.569)
FIB-4 index	—	124/422 (29.4)	0.74 (0.688-0.797)	0.628 (0.527-0.696)
APRI	—	124/422 (29.4)	0.717 (0.668-0.773)	0.558 (0.468-0.646)
SAFE score	—	124/422 (29.4)	0.791 (0.738-0.829)	0.65 (0.534-0.719)
LiverRisk score	≥14	72/422 (17.1)	0.738 (0.683-0.795)	0.326 (0.233-0.395)
FIB-4 index	—	72/422 (17.1)	0.779 (0.712-0.838)	0.542 (0.422-0.672)
APRI	—	72/422 (17.1)	0.751 (0.68-0.813)	0.465 (0.335-0.589)
SAFE score	—	72/422 (17.1)	0.818 (0.753-0.873)	0.547 (0.42-0.649)

Abbreviations: APRI, AST to Platelet Ratio Index; AUPRC, area under the precision-recall curve; FIB-4, fibrosis-4 index; MASLD, metabolic dysfunction–associated steatotic liver disease; NHANES, National Health and Nutrition Examination Surve; SAFE, steatosis-associated Fibrosis Estimator.

While LiverRisk score still had consistently higher AUROC and AUPRC than FIB-4 index and APRI among participant subsets, the differences were smaller than among all participants (Table S5, http://links.lww.com/HC9/B16). Further, differences in AUROC between LiverRisk score, FIB-4 index, and APRI were not statistically significant for any of the 3 classification tasks among participants who were non-Hispanic Black, Hispanic, who had type 2 diabetes, who reported daily alcohol consumption, or who did not have steatosis (Supplemental Tables S6, http://links.lww.com/HC9/B16). In contrast, SAFE score still significantly outperformed FIB-4 index in AUROC among participants who were non-Hispanic Black, Hispanic, or who had type 2 diabetes for the ≥8 kPa classification task. SAFE score also significantly outperformed LiverRisk score in AUPRC for at least 1 of the 3 classification tasks in all subsets analyzed except for non-Hispanic Asians.

We assessed threshold-dependent performance for each score while using F1 score, the harmonic mean of precision (positive predictive value) and recall (sensitivity), as an optimization metric (Supplemental Table S7, http://links.lww.com/HC9/B16). For the ≥8, ≥10, and ≥14 kPa classification tasks, F1 score was highest for LiverRisk scores of 7, 7, and 9; FIB-4 indices of 1.45, 2.5, and 3.25; APRI of 0.5, 0.5, and 0.75; and SAFE scores of 100, 150, and 250, respectively. However, real-world cutoff selection requires context of the use case, with each of these cutoffs resulting in a different tradeoff between precision and recall. For example, for the ≥8 kPa task, a LiverRisk score cutoff of 7 yields a precision of 0.294 and sensitivity of 0.345, while a SAFE score cutoff of 100 yields a precision of 0.416 and recall of 0.248 (Supplemental Table S7, http://links.lww.com/HC9/B16).

LiverRisk score and SAFE score also had significantly higher correlations with stiffness measurements than FIB-4 index or APRI, with Spearman coefficients of 0.31 (0.28–0.34), 0.28 (0.25–0.31), 0.11 (0.08–0.14), and 0.17 (0.14–0.20), respectively (Supplemental Figure S2, http://links.lww.com/HC9/B17). However, as these metrics primarily reflect accurate stratification of normal fibrosis measurements, they are less relevant to clinical practice than the classification metrics.

### Fibrosis prediction among participants with MASLD

Among NHANES participants with MASLD, LiverRisk score significantly outperformed FIB-4 index for the ≥8 kPa classification task in AUROC and AUPRC, but not among participants who were not non-Hispanic White, who had type 2 diabetes, or who had less than daily alcohol consumption (Tables S8, S9,http://links.lww.com/HC9/B16). For the ≥10 and ≥14 kPa classification tasks, LiverRisk score did not have a significantly different performance compared to either FIB-4 index or APRI, either among all participants or among participant subsets. While SAFE score outperformed FIB-4 index and APRI in both AUROC and AUPRC among all participants, there were no significant differences among participants who had type 2 diabetes or reported no alcohol consumption.

Among MASLD Registry participants, there were no significant differences in AUROC among any of the 4 scores, either among all participants or within participant subsets (Tables S10, S11, http://links.lww.com/HC9/B16). In contrast to both NHANES cohorts, both FIB-4 index and SAFE score outperformed LiverRisk score in AUPRC for all 3 classification tasks, including among Hispanic participants.

Cutoffs for optimizing F1 score were lower among both cohorts compared to the NHANES (all) cohort. Among NHANES participants with MASLD, for the ≥8, ≥10, and ≥14 kPa classification tasks, F1 score was highest for LiverRisk scores of 6, 6, and 9; FIB-4 indices of 1, 1.3, and 2.05; APRI of 0.25, 0.25, and 0.5; and SAFE scores of 100, 100, and 200, respectively (Supplemental Table S12, http://links.lww.com/HC9/B16). For the same tasks among MASLD Registry participants, F1 score was highest for LiverRisk scores of 6, 7, and 7; FIB-4 indices of 1, 1.9, and 1.9; APRI of 0.25, 0.25, and 0.5; and SAFE scores of 50, 150, and 200, respectively (Supplemental Table S13, http://links.lww.com/HC9/B16).

All 4 scores had significantly higher correlations with liver stiffness in the MASLD Registry than in the NHANES (MASLD) cohort (Supplemental Figure S2, http://links.lww.com/HC9/B17). In the former, there were no significant differences between correlations of the 4 scores, while in the latter, LiverRisk score, APRI, and SAFE score all had higher correlations than FIB-4 index. In contrast to the Rotterdam and LiverScreen cohorts where LiverRisk score overestimated stiffness, LiverRisk score underestimated stiffness for 242 (57.3%) and 445 (53.7%) of the participants in the MASLD Registry and NHANES (MASLD) cohorts, respectively, among whom the median errors were −1.56 and −3.20 kPa.

## DISCUSSION

In this study, we evaluated the ability of LiverRisk score, FIB-4 index, APRI, and SAFE score to perform prognostication and fibrosis prediction among participants from the Mount Sinai Health System and the NHANES 2017–2020 cycle. For prognostication among participants without prior liver disease diagnoses, LiverRisk score was significantly associated with future liver-related hospitalization, liver outcomes, and cirrhosis, but there was no evidence of its superiority to FIB-4 index or APRI in predicting these outcomes when comparing normalized inputs, either among all participants or among subsets. For fibrosis prediction in the general population, while LiverRisk score and SAFE score outperformed FIB-4 index and APRI among all participants, only SAFE score did so consistently among participant subsets, whereas LiverRisk score underperformed among participants who were not non-Hispanic White or who had type 2 diabetes. For fibrosis prediction among participants with MASLD, SAFE score outperformed FIB-4 index and APRI in some comparisons, but none of the scores consistently outperformed all others. Cutoffs for all 4 scores among these participants that optimized precision and recall were lower than those among the general population, likely due to increased fibrosis prevalence.

The nonsuperiority of LiverRisk score may be attributed to differences in demographic composition between our cohorts and the derivation cohort: for prognostication, we observed significantly lower HRs per quintile increase in LiverRisk score for any liver outcome among women compared to men and among those not identifying compared to those identifying as non-Hispanic White, whereas HRs for FIB-4 index and APRI were not significantly different. In the general NHANES cohort, LiverRisk score did not significantly outperform FIB-4 index and APRI in most participant subsets. Additionally, many participants from the derivation cohort were recruited from either primary care settings or the general population,^12,13^ and thus may have had fewer comorbidities and lower rates of undiagnosed liver disease than participants receiving care at Mount Sinai or those with diagnosed MASLD. Considering this, LiverRisk score may benefit from recalibration for non-European populations and nonprimary care settings, as our multivariable Cox regressions in MSDW demonstrate that the additional features present in LiverRisk score (glucose, cholesterol, GGT) are significantly associated with liver outcomes even in the presence of FIB-4 index or APRI.

Interestingly, although SAFE score was trained on only patients with MASLD, our results in the general NHANES cohort suggest that it is broadly applicable regardless of MASLD status. Even among participants without steatosis in this cohort, SAFE score still outperformed all other scores in AUPRC for at least one of the classification tasks. Unlike LiverRisk score, SAFE score was trained using a diverse set of participants with histologically assessed fibrosis, which may explain its consistent performance across data sets and participant subsets.

This study has several limitations. First, our prognostication evaluation in MSDW was retrospective and included only participants with all 6 LiverRisk score measurements available. Given that AST, ALT, and GGT are not routinely ordered measurements, this likely selected for participants being evaluated for liver disease. In contrast, these measurements were uniformly collected for the LiverRisk score derivation and validation cohorts, creating differences in participant ascertainment. Nevertheless, 69% of MSDW participants had ALT measurements within normal ranges (<33 U/L for men and <25 U/L for women), and we did not observe significant differences in performance between the 47% with and 53% without nonliver comorbidities for LiverRisk score, FIB-4 index, or APRI, suggesting our results remain relevant for general patient populations. Further, LiverRisk score is intended for “general use in clinical practice,” including population screening of patients with chronic conditions,^3^ and many clinicians may generate LiverRisk score predictions using pre-existing measurements rather than obtaining new measurements; thus, our study may still be representative of real-world applications. Second, we could not compare liver cancer and mortality prognostication of the four scores due to low incidence and limited availability of biomarker-linked liver-specific mortality data outside of the UK Biobank, respectively. While Liu et al^14^ demonstrated that LiverRisk score predicted diabetes-specific mortality in the NHANES III cohort, liver outcome and liver-specific mortality data are unavailable for this cohort. With liver cancer being a top 5 cause of cancer death globally^15^ and with liver-specific mortality generally reflecting liver disease severity, it remains necessary to evaluate these scores among large, diverse cohorts with these data available. Fourth, because FIB-4 index and APRI are routinely used in the diagnosis and management of MASLD, the MASLD Registry may be subject to selection bias for participants with high values of these scores. Addressing this, we created a subcohort of NHANES participants with MASLD using objective controlled attenuation parameter scores to avoid this bias. Fifth, we observed large confidence intervals for AUROC and AUPRC when analyzing fibrosis prediction among participant subsets in the MASLD Registry, likely due to the small size of our cohort (422 participants); thus, our study may not have sufficient statistical power to detect differences in predictor performance between subsets.

Together, our results suggest that first, given the differences in results between our cohorts and the original validation cohorts, additional replication studies and possibly recalibration are needed before LiverRisk score can be widely adopted, particularly in health care settings with large non-European populations. Second, FIB-4 index and APRI will likely remain relevant in the clinical setting, especially when glucose, cholesterol, and GGT measurements are unavailable. Among patients with MASLD, FIB-4 index is easier to calculate than SAFE score as it does not require body mass index or globulin, and we did not consistently observe significant differences in AUROC or AUPRC between the 2 scores. Third, more accurate blood-based predictors of liver fibrosis are still needed, especially among patients with MASLD and other chronic liver diseases. These predictors should ideally be derived using large, diverse cohorts with histologically assessed fibrosis.

## Supplementary Material

## Data Availability

Further information about the MSDW and MASLD/MASH Registry data sets is available at https://labs.icahn.mssm.edu/msdw/ and https://icahn.mssm.edu/about/departments/medicine/research-office/medicine/nash-program, respectively. Data from the NHANES 2017–2020 cycle is accessible at https://wwwn.cdc.gov/nchs/nhanes/search/datapage.aspx?Component=Examination&Cycle=2017-2020. Robert Chen: conceptualization, data curation, formal analysis, investigation, methodology, visualization, writing—original draft, writing—review and editing; Ben Omega Petrazzini, Girish Nadkarni, and Ghislain Rocheleau: formal analysis, methodology, writing—review and editing; Meena B. Bansal: conceptualization, investigation, project administration, supervision, writing—review and editing; Ron Do: conceptualization, funding acquisition, investigation, project administration, supervision, writing—review and editing. This work was supported in part through the Mount Sinai Data Warehouse (MSDW) resources and staff expertise provided by Scientific Computing and Data at the Icahn School of Medicine at Mount Sinai. Robert Chen is supported by the National Institute of General Medical Sciences of the NIH (T32-GM007280). Ron Do is supported by the National Institute of General Medical Sciences of the NIH (R35-GM124836). Ron Do reported being a scientific co-founder, consultant and equity holder for Pensieve Health (pending) and being a consultant for Variant Bio, all not related to this work. Meena B. Bansal receives grant support from Pfizer, Siemens, The Kinetix Group, and Histoindex and serves as a consultant for Madrigal, Intercept, Fibronostics, NOVO Nordisk, GSK, Merck, Boston Pharma, and The Kinetix Group. Girish Nadkarni consults, advises, owns stock, and is employed by Renalytix. He consults, received grants, owns stock, and is employed by Heart Test Laboratories. He consults, advises, owns stock, and is employed by Pensieve Health. The remaining authors have no conflicts to report.
