# Supplementary material for: Comparison of blood-based liver fibrosis scores in the Mount Sinai Health System, MASLD Registry, and NHANES 2017–2020 study
Source: Hepatol Commun. 2024 Aug 26;8(9):e0515. doi: 10.1097/HC9.0000000000000515 (PMC11357697; doi:10.1097/HC9.0000000000000515)

**Figure S1.** Kaplan-Meier curves for FIB-4 index and APRI among 32,828 MSDW participants.


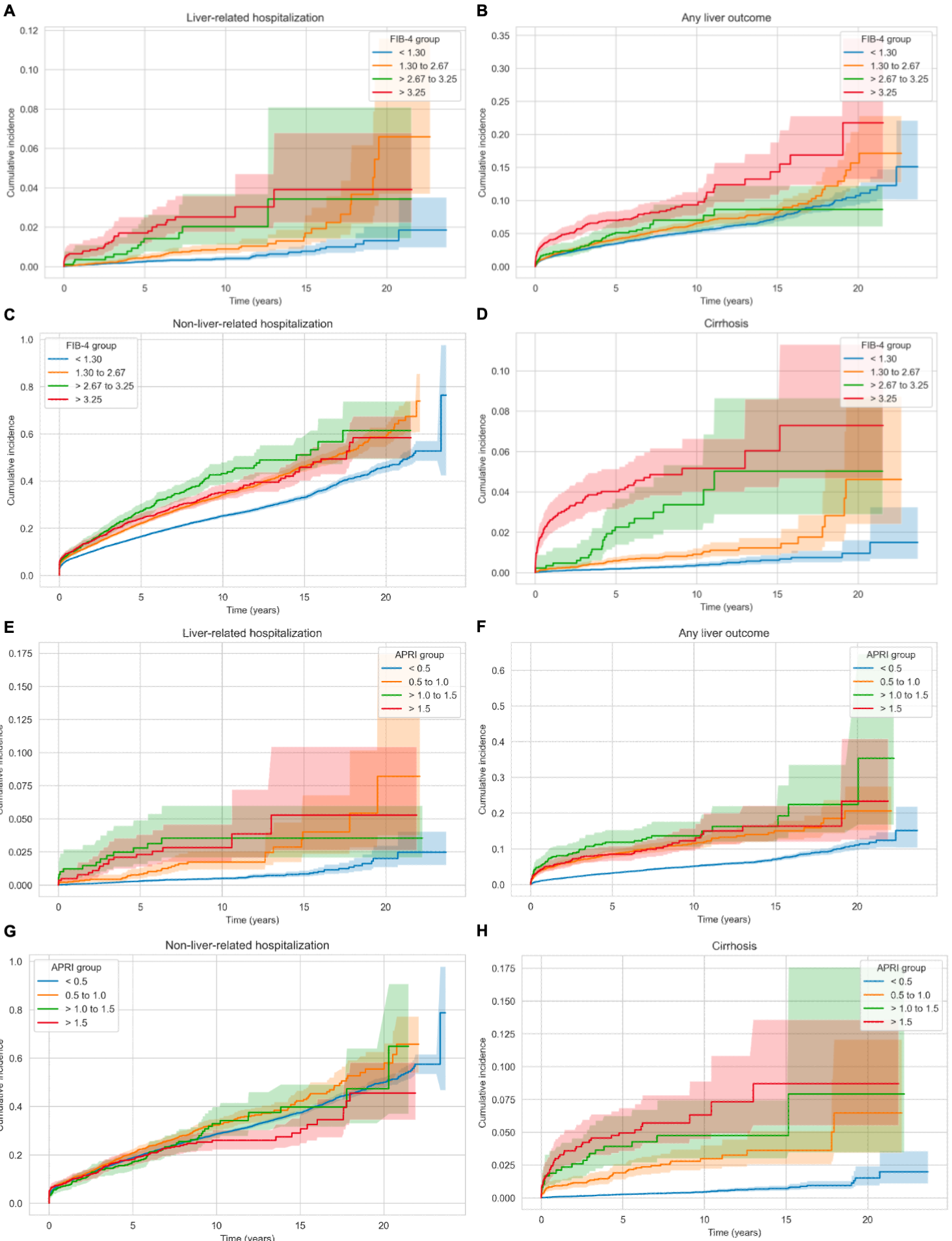


Kaplan-Meier curves with 95% confidence intervals showing the cumulative incidence of liver-related hospitalization (A, E), any liver outcome (B, F), non-liver-related hospitalization (C, G), and cirrhosis (D, H) for participants in different risk groups of FIB-4 index (A-D) or APRI (E-H).

**Figure S2.** Correlations between predictors and measured liver stiffness in three cohorts.


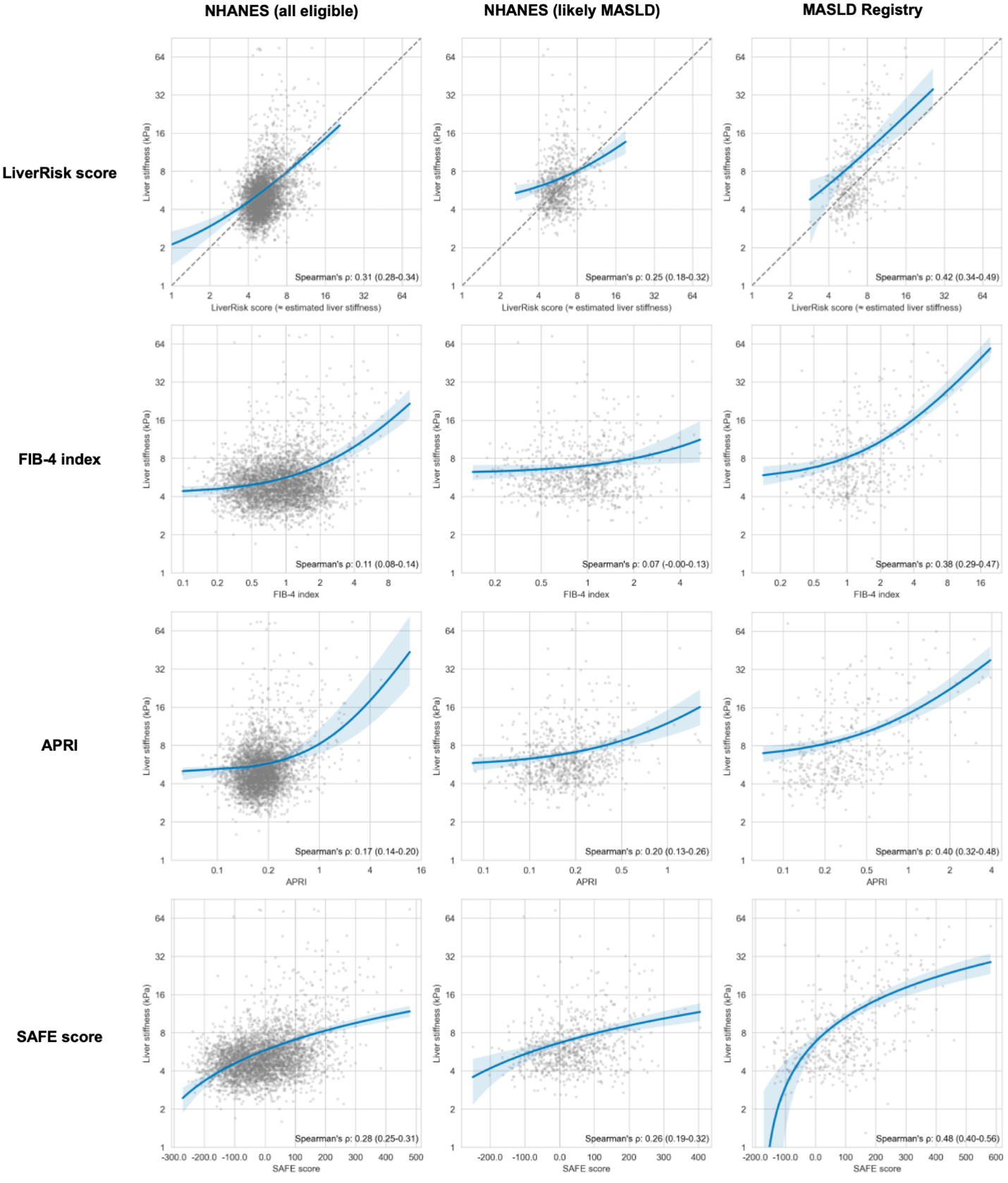

Supplement: Supplementary file 2 [file hc9-8-e0515-s002.docx]
